# Automated Segmentation of Cerebellum Using Brain Mask and Partial Volume Estimation Map

**DOI:** 10.1155/2015/167489

**Published:** 2015-04-28

**Authors:** Dong-Kyun Lee, Uicheul Yoon, Kichang Kwak, Jong-Min Lee

**Affiliations:** ^1^Department of Biomedical Engineering, Hanyang University, Hangdang-dong, Sungdong-gu, Seoul 133-791, Republic of Korea; ^2^Department of Biomedical Engineering, Catholic University of Daegu, Daegu, Gyeongsan-si 712-702, Republic of Korea

## Abstract

While segmentation of the cerebellum is an indispensable step in many studies, its contrast is not clear because of the adjacent cerebrospinal fluid, meninges, and cerebra peduncle. Thus, various cerebellar segmentation methods, such as a deformable model or a template-based algorithm might exhibit incorrect segmentation of the venous sinuses and the cerebellar peduncle. In this study, we propose a fully automated procedure combining cerebellar tissue classification, a template-based approach, and morphological operations sequentially. The cerebellar region was defined approximately by removing the cerebral region from the brain mask. Then, the noncerebellar region was trimmed using a morphological operator and the brain-stem atlas was aligned to the individual brain to define the brain-stem area. The proposed method was validated with the well-known FreeSurfer and ITK-SNAP packages using the dice similarity index and recall and precision scores. As a result, the proposed method was significantly better than the other methods for the dice similarity index (0.93, FreeSurfer: 0.92, ITK-SNAP: 0.87) and precision (0.95, FreeSurfer: 0.90, ITK-SNAP: 0.93). Therefore, it could be said that the proposed method yielded a robust and accurate segmentation result. Moreover, additional postprocessing with the brain-stem atlas could improve its result.

## 1. Introduction

It is well known that the human cerebellum is responsible for controlling the timing of various functional activities such as motor, balance, language, or distance measures [1–3]. It also supports cognitive processes such as emotion and attention [4, 5]. Therefore, cerebellar volume quantification using magnetic resonance imaging (MRI) has been widely used to investigate the cause of certain diseases, such as bipolar or motor disorders, to analyze cerebellar atrophy on traumatic brain injury, and to aid in the understanding of brain development with age [6, 7]. The delineation of the cerebellum is also important because it can be used as the reference region for intensity normalization of PIB or FDG PET analysis [8, 9].

Several methods for segmentation of the cerebellum, ranging from manual to automated, have been suggested [10, 11]. While manual delineation has been adopted and accepted as a gold standard, it suffers from inter- and intrarater variability, since it is tedious and time consuming [12–14]. Two distinct automated methods have been proposed to solve these problems: representative-deformable models and template-based approaches. Various representative-deformable models, such as active contour [10, 15], gradient vector flow [16], and level set, have been suggested since they are robust and unaffected by noise. Because the particular energy function, including intensity difference and sharpness, generally determines the resultant boundary, ambiguous boundaries and complex textures might trap the function. On the other hand, template-based approaches are based on a nonlinear registration algorithm [17–19] that computes the transformation from the reference volume to the target volume. Template-based approaches are the most widely used methods for brain segmentation, especially for lobar parcellation, since they have less constrained topology and can be applied to multiple segmentations simultaneously. However, these approaches might present the risk of systematic error in anatomical labeling due to the relatively high variability of individual cerebellar structures.

Recently, it has been suggested that combined conventional segmentation algorithms, such as deformable models or template-based methods and tissue classification algorithms, might overcome several limitations of the methods described in the previous paragraph. Ségonne et al. (2004) [20] presented a skull-stripping procedure combining the deformable model approach and a watershed algorithm. Kim et al. (2012) [21] constructed a deformable parametric model for the hippocampus from seed features obtained from multiple templates. Firbank et al. (2008) [22] integrated the template-based approach with tissue classification for the segmentation of the hippocampus. Shan et al. (2005) [11] proposed combining the advantages of both template- and deformable-model-based approaches, where the cerebellar template was chosen as a seed for the active contour. These approaches showed better segmentation results than any single method, especially when applied to complex structures.

Although all these approaches exhibit relatively accurate segmentation results, there are several obstacles to delineating the cerebellum exactly. For example, the surrounding structures of the cerebellum, such as the cerebellar peduncle, brain stem, and venous sinuses, have a similar intensity to the cerebellum itself. In the case of the venous sinuses, their boundaries and the cerebellum are divided into the thin cerebrospinal fluid (CSF). According to partial volume effects and intensity inhomogeneity, segmentation errors occurred in several methods [23]. Furthermore, the brain stem is connected to the cerebellum through the cerebellar peduncle, and there is no difference in their intensities.

In this study, we propose a fully automated method for segmentation of the cerebellum that combined tissue classification, a template-based approach, and morphological operations sequentially. The method was validated by comparing the results with the manual segmentation results of the LONI Probabilistic Brain Atlas (LPBA40) dataset [18] using the dice similarity index and recall and precision measures. The method was also compared with other popular packages such as FreeSurfer ([24], http://surfer.nmr.mgh.harvard.edu/) and ITK-SNAP ([35], http://www.itksnap.org).

## 2. Methods

### 2.1. Dataset

The LPBA40 dataset was used for validation of the proposed method ([18], http://www.loni.usc.edu/atlases/Atlas_Detail.php?atlas_id=12). It consists of 40 T1-weighted brain MRI data (20 males and 20 females, 29.20 ± 6.30 years). The scans were acquired with a three-dimensional spoiled gradient echo sequence on a GE 1.5 T system as 124 contiguous 1.5 mm coronal slices. The acquisition parameters were repetition time, 10.0–12.5 ms; echo time, 4.22–4.5 ms; flip angle, 20°; field of view, 220 mm or 200 mm. Experienced raters parcellated all 40 brain datasets manually into 56 structures, including the cerebellum and brain stem.

### 2.2. Data Processing

**Figure 1:**
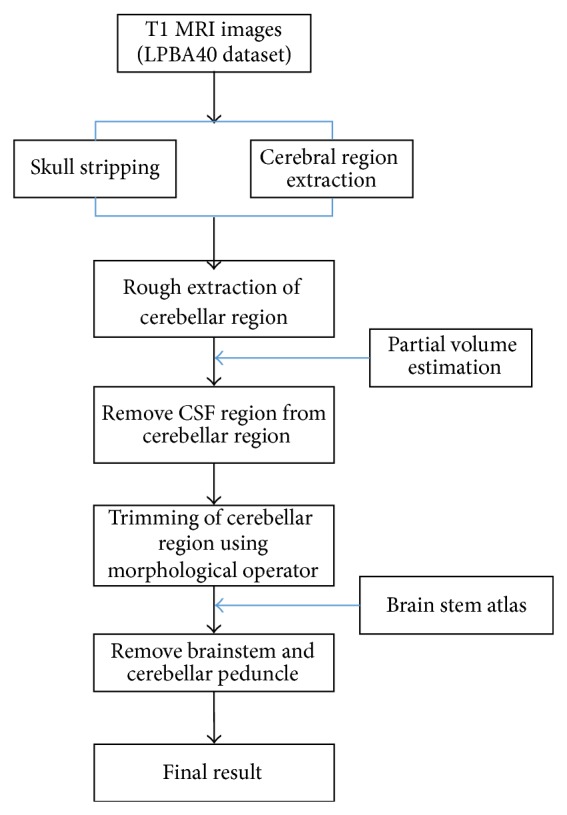
The flowchart of the proposed cerebellar segmentation method.

**Figure 2:**
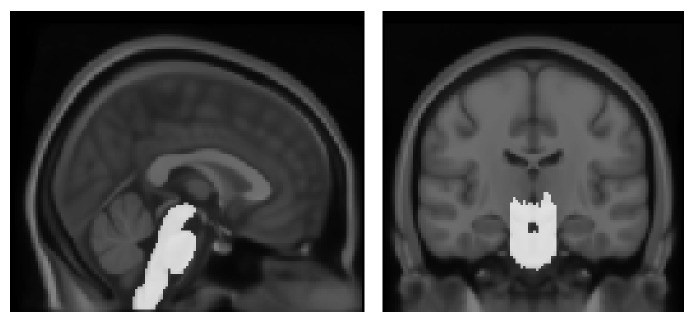
Brain-stem atlas image. The brain-stem atlas was defined manually on the International Consortium for Brain Mapping (ICBM) 152 template.

The proposed method for cerebellum segmentation consists of several consecutive steps (Figure 1). A preprocessing step includes intensity inhomogeneity correction, skull stripping, tissue classification, and partial volume estimation. We removed the cerebral region from the skull-stripped image to define the cerebellar region approximately and thresholded its partial volume image to remove the false positive. Morphological operators were then applied to eliminate the noise or nonconnected regions. The brain-stem template was generated by delineating the regions of brain stem and cerebellar peduncle manually on the International Consortium for Brain Mapping 152 (ICBM 152) template and aligned with the individual brain to remove the brain stem and cerebellar peduncle from the cerebellar region (Figure 2).

#### 2.2.1. Preprocessing and Extraction of Cerebellar Region

**Figure 3:**
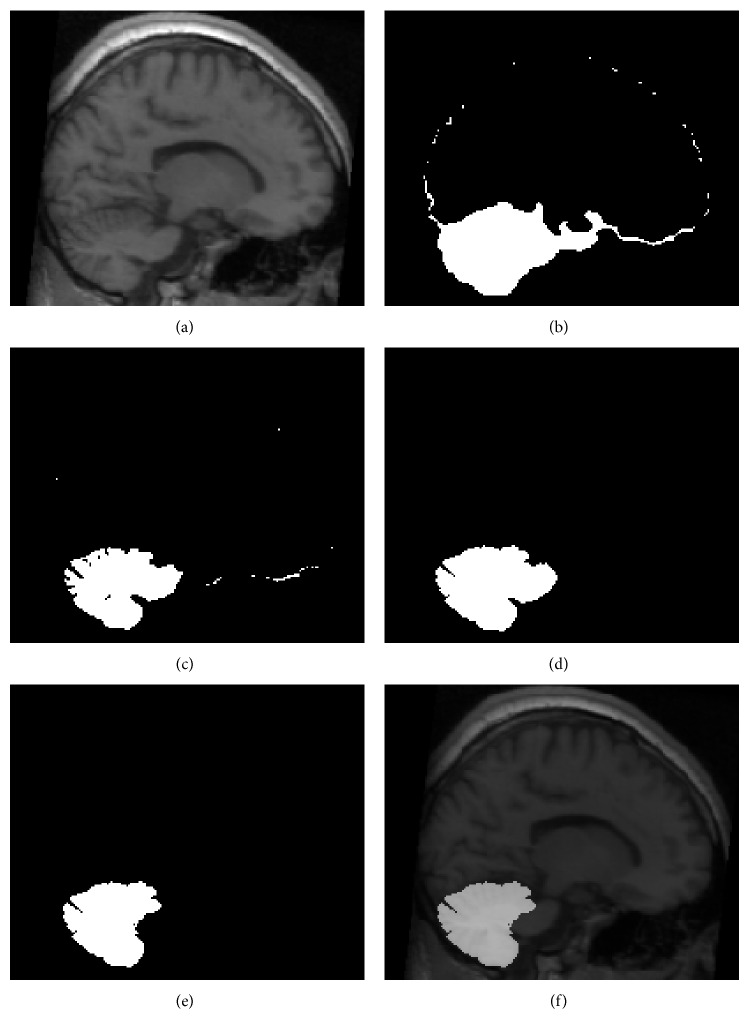
Snapshot of each step in the proposed segmentation procedure. (a) Individual T1 image. (b) Roughly defined cerebellar region using skull mask and brain mask. Cerebellar region candidate A. (c) Improvement in defining the cerebellar region via the CSF partial volume estimation map. (d) Trimming of the cerebellar region using a morphological operator. Cerebellar region candidate B. (e) Removal of brain stem and cerebellar peduncle using template-based segmentation. (f) Final result.

We corrected the intensity inhomogeneity, which involved varying the signal intensity slowly over the image caused by magnetic field inhomogeneity [25]. Skull stripping was performed using a Brain Extraction Tool (BET) that used a deformable model fitted to the brain surface using optimization parameters [26]. Each brain was transformed separately into a standardized stereotaxic space, that is, an ICBM 152 template, and resampled on a 1 mm^3^ voxel grid to account for interindividual differences in absolute brain size [27]. An artificial neural network classifier was applied to identify gray matter (GM), white matter (WM), and CSF [28]. Partial volume levels and MRI intensity mixing at the tissue interfaces due to the finite resolution of the imaging device were estimated and corrected using a trimmed minimum covariance determinant method [29]. A cortical surface was extracted automatically from each MR volume using the Constrained Laplacian-based Automated Segmentation with Proximities (CLASP) Algorithm to describe the cerebral region without the cerebellum [30]. The cerebellar region was then defined approximately by subtracting the cerebral region generated by the cortical surface from the skull-stripped volume. This region was called cerebellar region candidate A (Figure 3(b)).

#### 2.2.2. Morphological Operation and Template-Based Segmentation

It is important to separate the cerebellar tissue from nearby structures, such as the venous sinuses, cerebellar peduncle, and brain stem, which have a similar intensity of cerebellum. Because a thin CSF region divided the venous sinuses from the cerebellum, they were hard to be separated accurately because of the partial volume effect which was the amount of each tissue type within each voxel. Therefore, thresholding of the partial CSF volume image followed by morphological erosion was performed to remove the venous sinuses from the previously defined cerebellar region candidate A. A connected component analysis was then applied to select the largest region as the true positive cerebellar region. We defined this region as cerebellar region candidate B. Finally, morphological dilation restored cerebellar region candidate B to its original size (Figures 3(c) and 3(d)).

Since the brain stem is connected directly to the cerebellum, it was not removed completely in the previous step. The template-based approach was applied to separate the brain stem from cerebellar region candidate B. The brain stem template was delineated manually on the ICBM 152 atlas which was generated by averaging anatomical MRI data of 152 healthy normal adults corrections for overall brain size and orientation. It was aligned to each subject using nonlinear registration to mask out the brain stem and cerebellar peduncle from cerebellar region candidate B (Figures 3(e) and 3(f)). Since noise might have been introduced inadvertently during the masking of the brain stem and cerebellar peduncle, a morphological opening operation, a serial combination of erosion and dilation, was applied to remove any noise from the final result.

### 2.3. Validation

FreeSurfer assigns a neuroanatomical label automatically to each voxel of an individual MRI volume based on probabilistic information estimated from a manually labeled training set, and ITK-SNAP provides semiautomated segmentation using an active contour algorithm. In FreeSurfer, the “recon-all” command performed the intensity normalization, talairach registration, and labeling processes. In ITK-SNAP, the manually defined cerebellar region on the ICBM 152 template was registered to each subject using an affine transform and filled by an active contour algorithm. Differences between the gold standard and the segmentation results from FreeSurfer, ITK-SNAP, and the proposed method were examined with a paired *t*-test.

The dice similarity index and precision and recall measures were used for evaluating the proposed method. The dice similarity index, one of the most common methods for evaluating segmentation results, indicates a level of similarity between the reference and segmented volumes [31]:

(1) $$\begin{array}{c} \text{Dice Similarity Index}=\frac{V_{m}\cap V_{a}}{\left(V_{m}+V_{a}\right)/2}, \end{array}$$

where *V*_*m*_ and *V*_*a*_ are the voxel sets segmented as cerebellum in the manual delineation and each method, respectively. They range from 0 for sets that have no common elements to 1 for identical sets. The precision is the number of true positives (i.e., the number of items labeled correctly as belonging to the positive class) divided by the total number of elements labeled as belonging to the positive class (i.e., the sum of true positives and false positives). The recall is defined as the number of true positives divided by the total number of elements that actually belong to the positive class (i.e., the sum of true positives and false negatives) [32]. They are defined as follows:

(2) PrecisionVm∩VaVa,Recall=Vm∩VaVm.

A precision score of 1.0 for class *V*_*m*_ means that every item labeled as belonging to class *V*_*m*_ belongs to class *V*_*a*_ but says nothing about the number of items from class *V*_*m*_ that are labeled incorrectly. On the other hand, a recall of 1.0 means that every item from class *V*_*m*_ is labeled as belonging to class *V*_*a*_ but says nothing about how many other items are incorrectly labeled as also belonging to class *V*_*m*_.

## 3. Result

### 3.1. Qualitative Evaluation of Similarity and Comparison with FreeSurfer and ITK-SNAP

**Figure 4:**
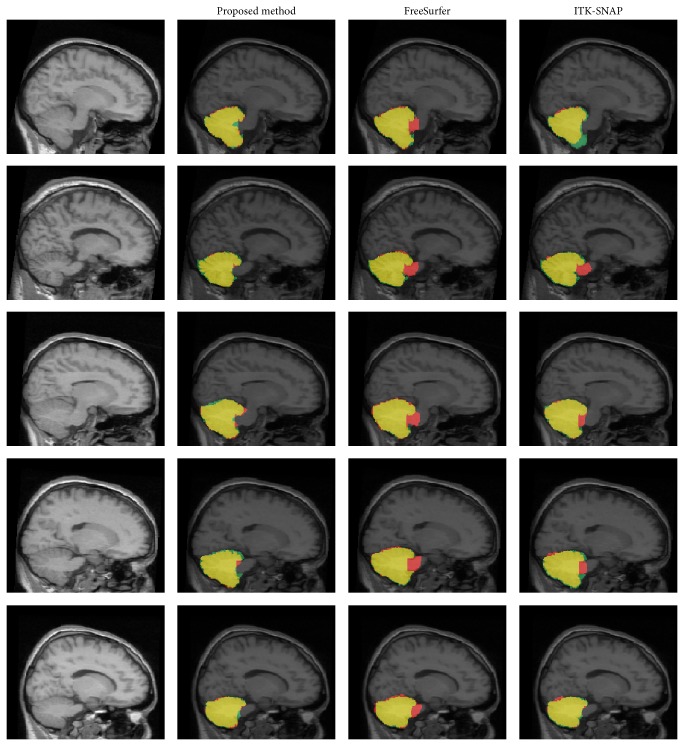
Comparison of each automatic segmentation method and manual definition. Yellow is an identical result; red is overestimation; green is underestimation.

**Figure 5:**
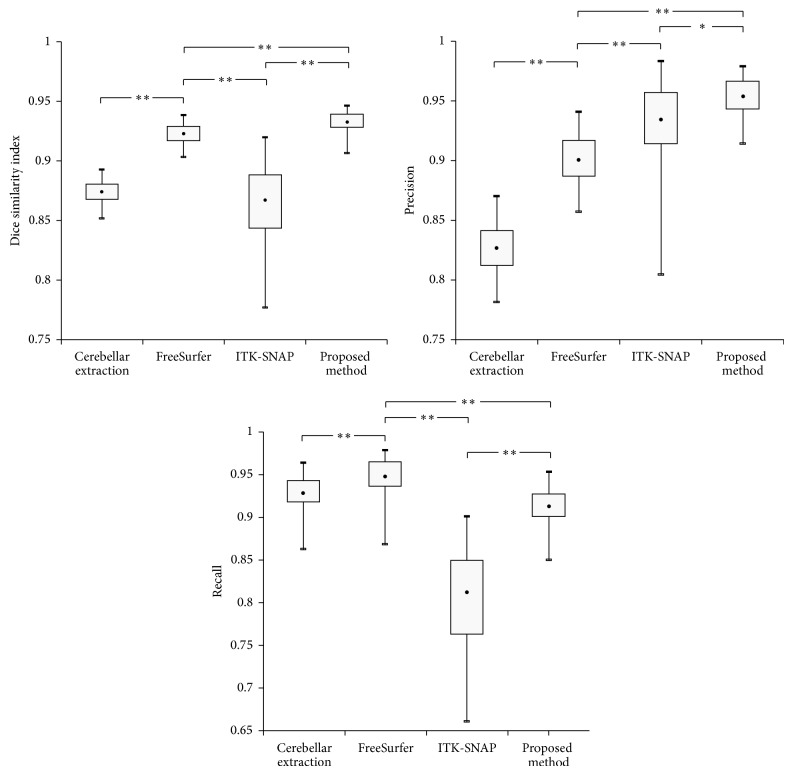
Box plot of quantitative indices (^∗∗^ *p* < 0.001, ^∗^ *p* < 0.01, where *p* indicates the statistical significance).

Figure 4 shows the segmentation results from FreeSurfer, ITK-SNAP, and the proposed method, and their differences from the manual gold standard. While FreeSurfer and ITK-SNAP exhibited over- or underestimated results, the proposed method showed better results, mainly from the removal of the brain stem.  Figure 5 shows the dice similarity index for each method. The proposed method showed a significantly higher dice similarity index (0.932 ± 0.008) than FreeSurfer (0.923 ± 0.009) and ITK-SNAP (0.867 ± 0.033). This meant that the proposed method performed better than the other methods in terms of likeness.

We compared the results before and after removing the brain stem to investigate the effect of the template-based segmentation on the proposed method. The dice similarity index improved significantly after processing, as expected (0.874 ± 0.009 versus 0.932 ± 0.008). For a better understanding, it was also applied to the results of FreeSurfer and ITK-SNAP. Even though there was no significant difference, indices for both methods increased relatively (FreeSurfer: 0.929 ± 0.009; ITK-SNAP: 0.879 ± 0.033) after removing the brain stem.

### 3.2. Qualitative Evaluation of Precision and Recall and Comparison with FreeSurfer and ITK-SNAP

**Figure 6:**
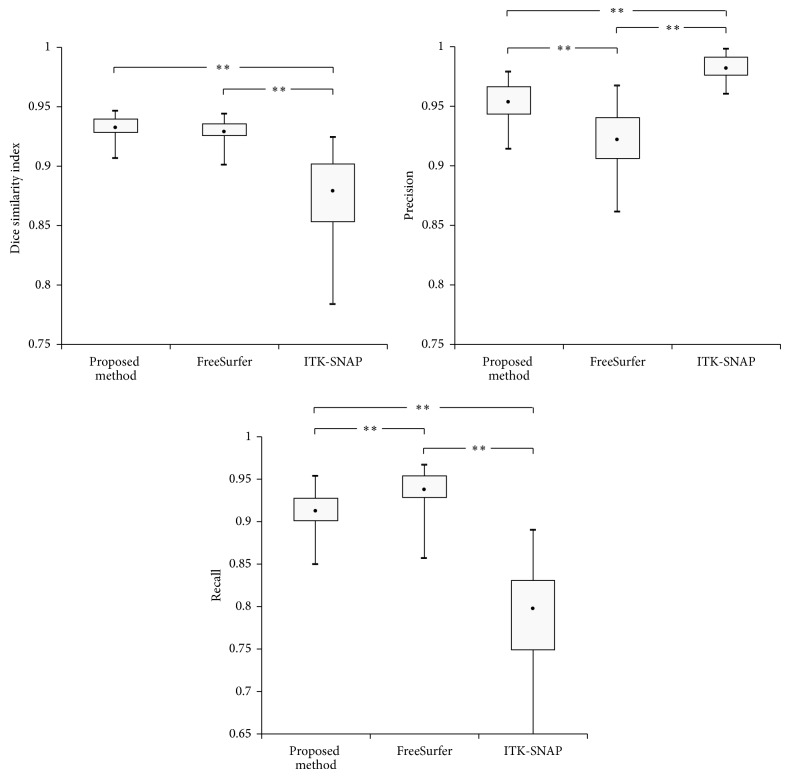
The box plot of quantitative indices after removing the brain stem and cerebellar peduncle (^∗∗^ *p* < 0.001, where *p* indicates the statistical significance).

Figure 6 shows differences in the recall and precision values among the three approaches. FreeSurfer exhibited significantly higher recall values (0.948 ± 0.023) than ITK-SNAP (0.812 ± 0.056) and the proposed method (0.913 ± 0.023), while the proposed method showed significantly higher precision values (0.953 ± 0.017) than FreeSurfer (0.900 ± 0.022, *p* < 0.0001) and ITK-SNAP (0.934 ± 0.037, *p* = 0.004).

When the template-based segmentation of the brain stem was applied, the recall value decreased but the precision value increased after removing the brain stem from all the methods (Table 1).

## 4. Discussion

In this study, we propose a fully automated framework for cerebellum segmentation that consists of tissue classification, cortical surface extraction, template-based segmentation, and morphological operations. The segmentation results of the proposed method were compared with FreeSurfer and ITK-SNAP, which are widely employed in brain segmentation. The LPBA40 dataset with a manually defined cerebellum was used as the gold standard for an objective and righteous validation.

While the segmentation results seemed to show a relatively well-defined boundary for the cerebellum, the robustness and accuracy of each method were demonstrated using the quantitative evaluations of the dice similarity index, and recall and precision values. As described in Section 3, the proposed method showed better performance in the dice similarity index than FreeSurfer and ITK-SNAP. Most segmentation errors occurred in the cerebellar peduncle and venous sinuses, since the intensity of the cerebellar peduncle was very similar to that of the cerebellar WM. FreeSurfer tended to fail in removing the cerebellar peduncle and venous sinuses exactly [10]. This was because the atlas in FreeSurfer excluded the venous sinuses and it could not distinguish accurately between the brain stem and cerebellum (Figure 4) [10, 23, 33, 34]. On the other hand, ITK-SNAP tended to underestimate the cerebellar surface compared with manual delineation (Figure 4). ITK-SNAP is a semiautomatic approach using an active contour model where the seed point is extended to the image boundary. It showed relatively poor performance in the regions with inaccurate boundaries, since the propagation of the contour depends on an edge or curvature [35].

**Figure 7:**
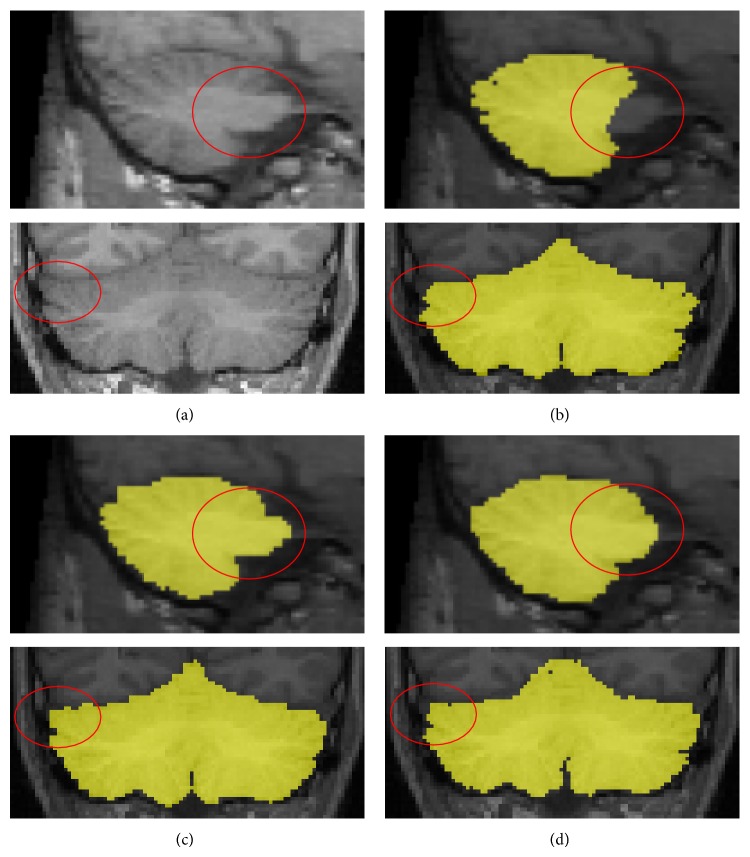
Comparison of the cerebellar segmentation performed with (b) proposed method (c), FreeSurfer, and (d) ITK-SNAP. Incorrect segmentation of venous sinuses (red circle) could be a problem when using the automated software.

Reliable separation of the brainstem and cerebellar peduncle from cerebellum is necessary to achieve an accurate measurement of cerebellar volume [23]. For this reason, we explored the effect of the brain stem including the cerebellar peduncle. After applying template-based segmentation for the brain stem to the results of FreeSurfer and ITK-SNAP, the cerebellar peduncle was separated more accurately than in the previous results and the similarity index and precision value were significantly enhanced (Figure 6 and Table 1). The difference in the similarity index between the proposed method and FreeSurfer or ITK-SNAP could be caused by incorrect discrimination of the cerebellar tissue from nearby structures, such as the venous sinuses (Figure 7). To avoid this problem, we eliminated the nonconnected region using a morphological operator and partial volume estimated images. As a result, our proposed method showed significantly higher indices of similarity than the other methods, even though they also removed the brain stem.

In conclusion, we propose a fully automated procedure for cerebellar segmentation including template-based segmentation and morphological operations. The proposed method showed accurate segmentation results when compared with manual delineation and removed the cerebellar peduncle from the cerebellum effectively.

## Figures and Tables

**Table 1:** Precision and recall indices before and after removing the brain stem.

	Recall		Precision	
	Before	After	Before	After
Proposed method	0.928 (0.0226)	0.913 (0.0234)	0.827 (0.0210)	0.953 (0.0172)^∗∗^
FreeSurfer	0.948 (0.0225)	0.937 (0.0227)	0.900 (0.0234)	0.922 (0.0230)^∗∗^
ITK_SNAP	0.812 (0.0562)	0.800 (0.0556)	0.934 (0.0366)	0.981 (0.0103)^∗∗^

^∗∗^ *p* < 0.001, where *p* indicates the statistical significance.
